# Whole-Genome Sequence of the Microcystin-Degrading Bacterium *Sphingopyxis* sp. Strain C-1

**DOI:** 10.1128/genomeA.00838-15

**Published:** 2015-07-30

**Authors:** Kunihiro Okano, Kazuya Shimizu, Hideaki Maseda, Yukio Kawauchi, Motoo Utsumi, Tomoaki Itayama, Zhenya Zhang, Norio Sugiura

**Affiliations:** a Department of Biological Environment, Faculty of Bioresource Sciences, Akita Prefectural University, Akita City, Akita, Japan; b Faculty of Life Sciences, Toyo University, Gunma, Japan; c Department of Biological Science & Technology, Institute of Technology and Science, Tokushima University Graduate School, Tokushima, Japan; d MC Evolve Technologies Corporation, Ibaraki, Japan; e Faculty of Life and Environmental Sciences, University of Tsukuba, Ibaraki, Japan; f Graduate School of Engineering, Nagasaki University, Nagasaki, Japan; g Malaysia-Japan International Institute of Technology, Universiti Technologi Malaysia, Kuala Lumpur, Malaysia

## Abstract

This report describes the whole-genome sequence of an alkalitolerant microcystin-degrading bacterium, *Sphingopyxis* sp. strain C-1, isolated from a lake in China.

## GENOME ANNOUNCEMENT

Harmful algal blooms (HABs) producing microcystin, a potent hepatotoxin, are a serious health problem worldwide regarding the safety of potable water. Microcystin is stable against heat, pH, and common proteases (1, 2) but can be degraded by specific enzymes, MlrA, MlrB, MlrC, and MlrD, which are encoded by a gene cluster of 4 genes, *mlrA*, *mlrB*, *mlrC*, and *mlrD*, respectively, in microcystin-degrading bacteria (2–6). MlrA, located in the inner membrane, is essential for initial microcystin degradation (7), MlrB degrades linearized microcystin, and MlrC degrades linearized microcystin and tetrapeptide (3–5). Alkalitolerant or alkaliphilic bacteria that can degrade microcystin, such as *Sphingopyxis* sp. strain C-1, play a key role in microcystin degradation within lakes and reservoirs with HABs, which generally have highly alkaline pH (8–10). Here, we report the whole-genome sequence of the microcystin-degrading bacterium *Sphingopyxis* sp. strain C-1 (9).

Whole-genome sequencing was carried out using an Illumina HiSeq 1000 system (Illumina, San Diego, CA, USA) with a paired-end library (400 bp) and an Illumina HiSeq 2500 system (Illumina) with a mate pair library (8 to 10 kb). A total of 245,219,908 reads, averaging 99 bp in length, were obtained, for a total of 2,422,368,447 bases of sequence. All reads were assembled *de novo* using Velvet version 1.2.08 (https://www.ebi.ac.uk/~zerbino/velvet/). Gaps between the resulting 33 contigs were closed using Platanus version 1.2.1 (http://platanus.bio.titech.ac.jp/platanus-assembler/). The whole genome was annotated using the RAST server (http://rast.nmpdr.org/rast.cgi), which predicted protein-coding sequences (CDSs).

The *Sphingopyxis* sp. strain C-1 genome was represented by 2 contigs, BBRO01000001 and BBRO01000002. PCR was used to detect their mutual orientation, which revealed that the 3′ end of BBRO01000001 was connected to the 5′ end of BBRO01000002, and the 3′ end of BBRO01000002 was connected to the 5′ end of BBRO01000001. The genome had a total length of 4,583,092 bp and a G+C content of 63.70%, with 4,318 CDSs and 48 RNA-coding genes (i.e., 16S, 23S, and 5S rRNA genes and 45 tRNA genes). The annotation revealed that 2,937 CDSs exhibited homology to genes with known functions, while the remaining 1,381 CDSs encoded hypothetical proteins with unknown functions.

The genes encoding MlrB, MlrD, MlrA, and MlrC of *Sphingopyxis* sp. strain C-1 were designated with locus_tag SC1_04306 to SC1_04309 (1352105 to 1356054) on BBRO01000002. In addition, 2 adjacent genes, locus_tag SC1_04305 of 1,140 bp and locus_tag SC1_04304 of 1,461 bp, encoding peptide-modifying dipeptidase and d-aminoacylase, respectively, were identified in the same direction. Since these genes belonged to peptide-modifying enzymes adjoined to the *mlrB* gene in a head-to-head arrangement, these 6 genes were very likely to construct the microcystin-degrading enzyme gene cluster. Therefore, we designated these genes *mlrE* (locus_tag SC1_04305) and *mlrF* (locus_tag SC1_04304).

### Nucleotide sequence accession numbers.

This whole-genome shotgun project has been deposited at DDBJ/EMBL/GenBank under the accession no. BBRO00000000. The version described in this paper is the first version, BBRO01000000.
